# Noma Beyond Childhood: Dual-Flap Reconstruction of Orofacial Necrotising Infection in an Immunocompromised Elderly Male Patient

**DOI:** 10.7759/cureus.111902

**Published:** 2026-07-01

**Authors:** Jevina Daphne Winnifred, Rohan Dsouza, Leo F Tauro

**Affiliations:** 1 General Surgery, Father Muller Medical College Hospital, Mangalore, IND

**Keywords:** cancrum oris, cheek reconstruction, immunocompromised, noma, orofacial necrosis

## Abstract

Cancrum oris (noma) is a rapidly progressive orofacial gangrenous infection predominantly described in malnourished children. Its occurrence in immunocompromised elderly adults is rare, diagnostically challenging, and associated with high mortality rates without timely intervention. We report a case that highlights the general surgical principles governing its recognition and management. A 73-year-old man presented with a one-month history of right cheek swelling, ulceration, and trismus. Examination revealed an indurated, ulcerated lesion with purulent discharge and necrotic margins. Serial surgical debridements were performed, guided by culture-directed antibiotic therapy and concurrent nutritional rehabilitation. Definitive reconstruction was achieved with a right buccal mucosal advancement flap and cheek rotational fasciocutaneous flap. This case reaffirms that noma must be considered in elderly immunocompromised patients presenting with orofacial necrosis. A structured surgical strategy that includes aggressive debridement, broad-spectrum antimicrobial therapy, nutritional optimization, and staged locoregional reconstruction is the determinant of survival and functional recovery.

## Introduction

Cancrum oris, synonymous with noma, derives from the Greek nomein, meaning “to devour." It's a term that aptly captures the destructive trajectory of this disease [1]. Noma is exceptionally rare, representing fewer than 5% of global cases. It is a fulminant, polymicrobial necrotizing infection that originates at the oral mucosa and relentlessly advances through the buccal fat, musculature, and overlying skin, producing full-thickness orofacial defects of devastating functional and cosmetic consequence. The global burden of noma is disproportionately carried by malnourished children aged between two and six years in sub-Saharan Africa, where poverty, inadequate sanitation, and undernutrition converge to produce susceptibility [2]. The condition was formally included in the World Health Organization (WHO) list of Neglected Tropical Diseases in 2023, reflecting its continued public health significance [3]. In high-income settings, however, noma is largely unrecognized, and its occurrence in immunocompromised adults-including those with HIV/AIDS, hematological malignancies, or immunosenescence of advanced age-is increasingly documented, frequently presenting atypically and thereby delaying diagnosis [4]. Reported mortality in untreated noma approaches 90% [5]. Even with treatment, survivors are left with severe disfigurement, trismus, and psychosocial sequelae. The general surgeon plays a pivotal role across all phases of management-from initial wound assessment and staged debridement to reconstructive planning and perioperative optimization. We present the case of a 73-year-old immunocompromised male patient who developed noma affecting the right cheek, managed successfully with serial debridement and locoregional reconstruction, with the aim of highlighting the diagnostic and surgical decision-making framework applicable to this uncommon but life-threatening condition.

## Case presentation

A 73-year-old male patient with a known case of uncontrolled type 2 diabetes mellitus and dyslipidemia for 15 years (HbA1c = 10.6%) on oral hypoglycemic and antihyperlipidemic drugs was referred to the Department of General Surgery with a one-month history of progressive swelling and ulceration of the right cheek, accompanied by worsening restriction of mouth opening. The patient reported no antecedent dental procedure, trauma, or previous similar episode. Associated symptoms included reduced oral intake, progressive weight loss over three months following the initial onset of symptoms (on admission serum albumin = 2.7 g/dl), and low-grade intermittent fever. There was no history of foreign body ingestion or intraoral instrumentation. Local examination of the orofacial region revealed a solitary indurated swelling over the right buccal region, measuring approximately 5 × 4 cm. The central zone demonstrated frank ulceration with undermined necrotic margins, surrounding erythema, and copious purulent discharge with a fetid odor. The overlying skin showed early dusky discoloration indicative of impending cutaneous involvement (Figures 1-2). Maximum inter-incisal opening (trismus) was restricted to 35 mm, which affected the patient's ability to speak clearly or eat comfortably. Ipsilateral submandibular lymphadenopathy was present. The lips, palate, floor of mouth, and contralateral cheek were uninvolved.

**Figure 1:**
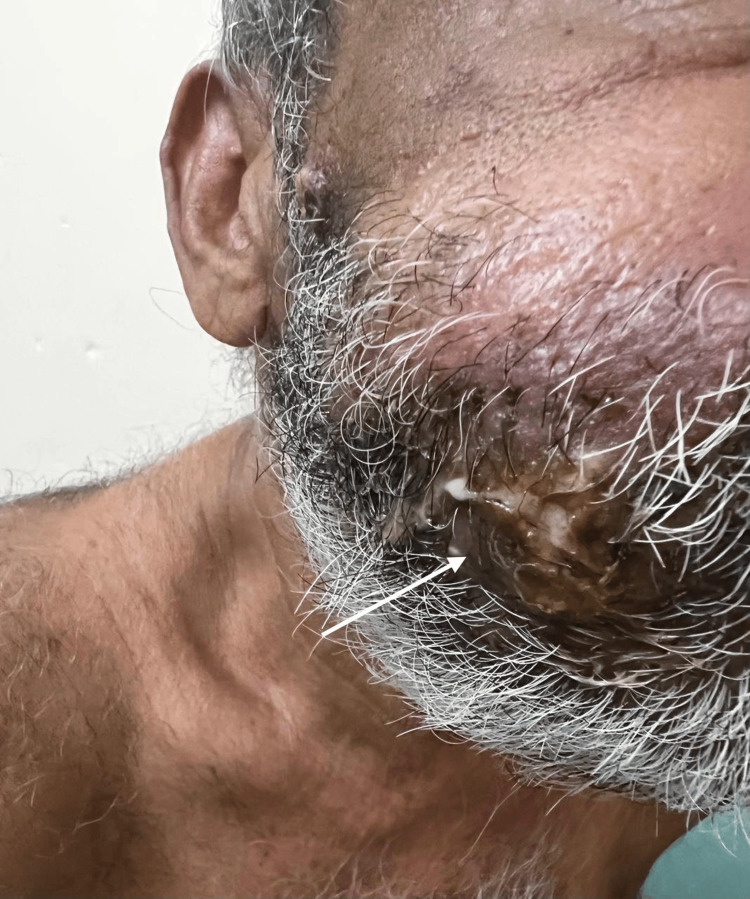
Preoperative clinical photograph demonstrating right-sided orofacial gangrene. The white arrow depicts the central necrotic ulceration with eschar, purulent discharge, and surrounding indurated erythema extending from the right buccal region to the preauricular area. The findings are consistent with established gangrenous noma in an immunocompromised elderly host.

**Figure 2:**
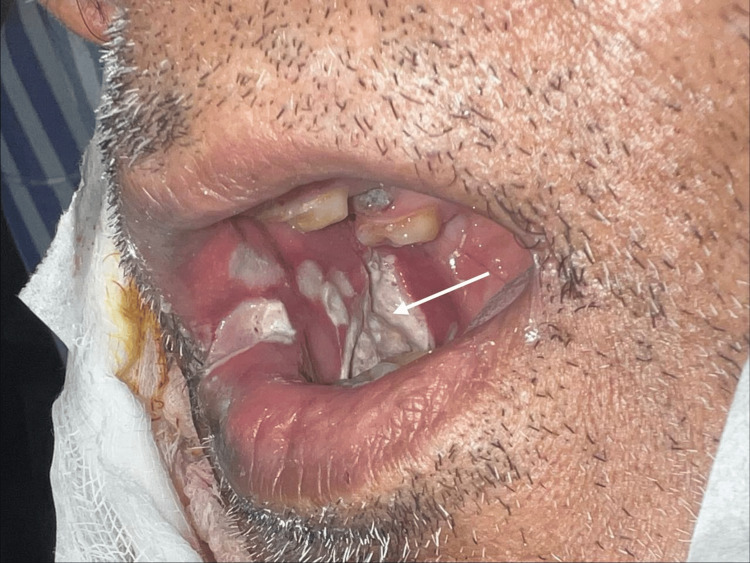
Intraoral photograph demonstrating necrotic sloughing of the right buccal mucosa (white arrow). The grey-white pseudomembranous necrotic plaque with surrounding mucosal erythema and edema confirms full-thickness transmural involvement of the right buccal wall. Poor dentition and restricted mouth opening (trismus) are also evident.

Investigations

Wound swab cultures obtained prior to antibiotic initiation yielded polymicrobial growth, including *Staphylococcus aureus*, *Staphylococcus epidermidis*, and the *Acinetobacter baumannii *complex, with sensitivities guiding subsequent antibiotic rationalization. Contrast-enhanced computed tomography (CECT) of the face and neck was performed, which confirmed asymmetric right-sided soft tissue thickening with infiltration of the buccal and masseteric spaces, obliteration of normal fat planes, and no evidence of bony erosion or carotid involvement (Figure 3).

**Figure 3:**
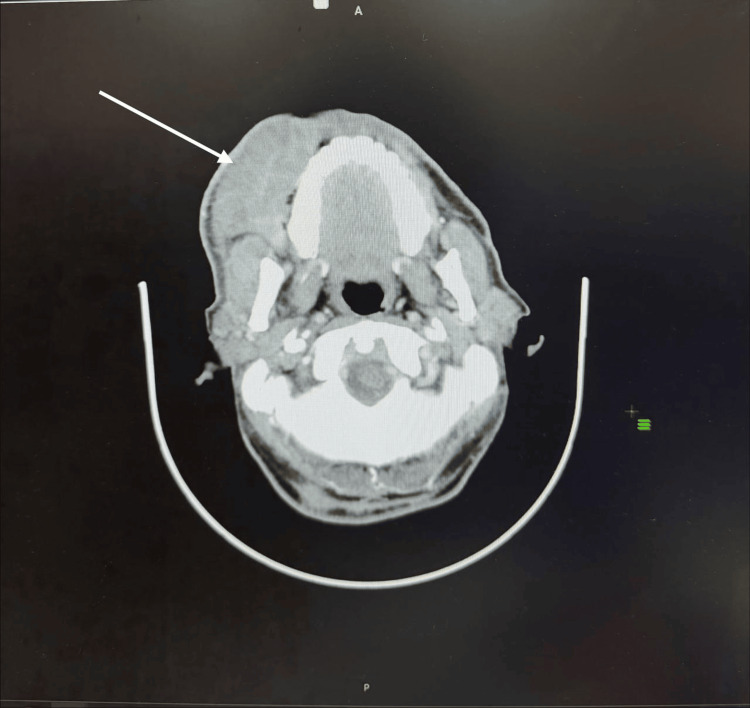
Axial contrast-enhanced CT of the neck demonstrating asymmetric right-sided soft tissue thickening and infiltration of the right buccal and masseteric space with obliteration of normal fat planes (white arrow), consistent with necrotising orofacial infection (cancrum oris). The oropharyngeal airway remains patent. No frank bony erosion or carotid vessel involvement is demonstrated on this cut.

Surgical management and postoperative course

On admission, the patient was resuscitated with intravenous crystalloid fluids. High-protein nutritional supplementation was commenced via nasogastric feeding given inadequate oral intake. Empirical intravenous antibiotic therapy was initiated immediately, comprising metronidazole (500 mg every eight hours) for anaerobic coverage and piperacillin-tazobactam for aerobic Gram-negative cover, subsequently rationalized to a culture-directed regimen [6]. The patient underwent a total of three staged debridements at approximately four- to five-day intervals over a two-week period. The first procedure addressed gross necrotic tissue and eschar; subsequent sessions allowed progressive excision following tissue demarcation, culminating in a clean, granulating wound bed suitable for reconstructive planning. At each sitting, wound cultures were obtained, and dressings renewed. Wound assessment confirmed progressive formation of healthy granulation tissue, resolution of purulence, and eventual microbiological clearance (Figures 4-5) [7]. Wound cultures yielded polymicrobial growth. *Staphylococcus aureus* and epidermidis were considered primary pathogens based on heavy growth, known virulence, and clinical correlation with the pattern of tissue destruction. Secondary organisms like the *Acinetobacter baumannii* complex were identified as secondary opportunistic pathogens in the context of immunosuppression. Pain was managed using a multimodal analgesic regimen escalating along WHO analgesic principles [8], with opioid analgesia in the immediate post-debridement period. Pain scores (the Wong-Baker FACES Pain Rating Scale [9] was used) progressively declined following wound stabilization. 

**Figure 4:**
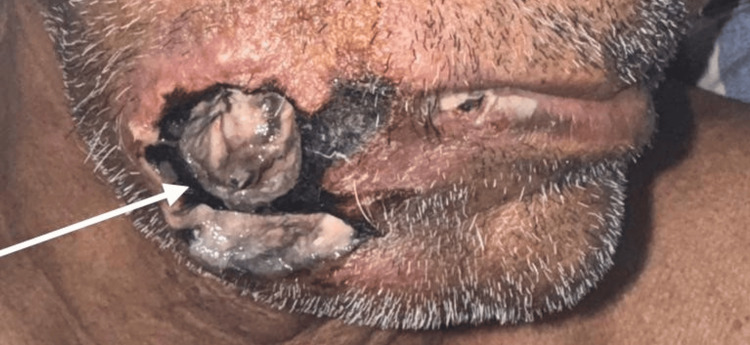
Frontal preoperative view demonstrating the established gangrenous eschar of cancrum oris affecting the right buccal region (white arrow).

**Figure 5:**
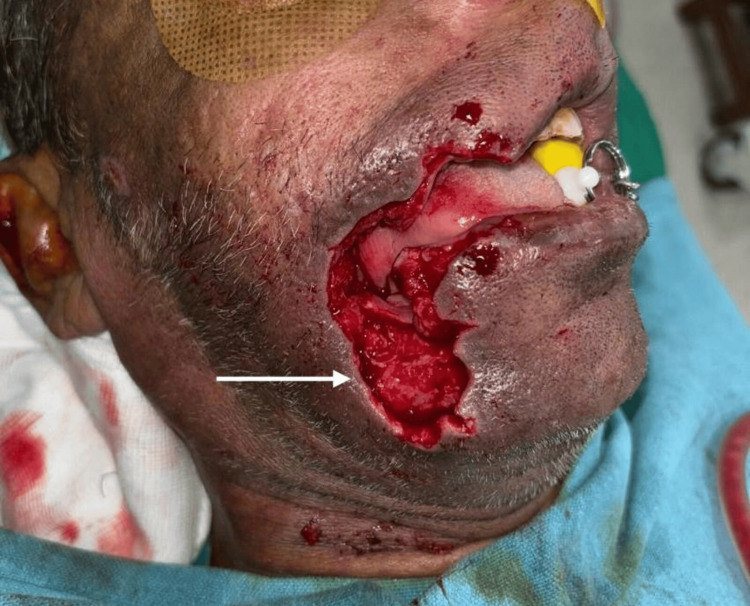
An intraoperative photograph following the final debridement (white arrow) demonstrating the full-thickness right buccal defect with actively bleeding viable wound margins.

Histopathological examination of tissue obtained at first debridement demonstrated extensive coagulative necrosis, dense acute and chronic inflammatory cell infiltrates, vascular congestion with thrombosis of small-caliber vessels, and the absence of malignancy, findings consistent with acute necrotizing fasciitis (orofacial necrotizing gangrene/noma), with no evidence of malignancy, fungal infection, or mycobacterial disease.

A definitive reconstructive plan was undertaken once the wound bed was deemed suitable, defined as complete granulation tissue coverage without residual slough or exposed bone. A two-layer reconstruction was performed. The intraoral mucosal defect was resurfaced with a right buccal mucosal advancement flap, mobilized from the posterior buccal mucosa and advanced anteriorly to reconstruct the defect (Figure 6). The external cutaneous defect was addressed with a cheek rotational fasciocutaneous flap, designed based on the facial artery perforator axis, and rotated to achieve tension-free coverage with tissue of comparable color and texture to the recipient site. Both flaps were inset with interrupted absorbable sutures for the mucosa and non-absorbable interrupted sutures for the skin (Figure 7) [10]. The fasciocutaneous flap was identified preoperatively by handheld Doppler and confirmed intraoperatively by visual identification of a pulsatile pedicle.

**Figure 6:**
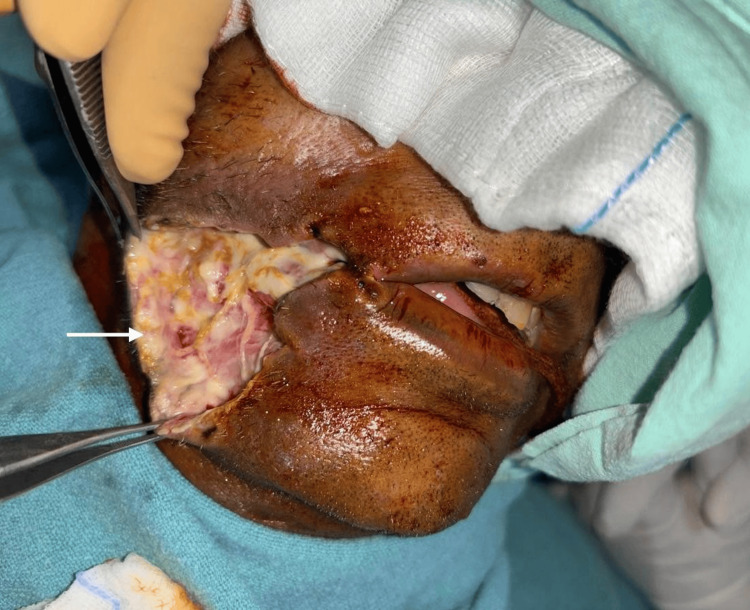
Intraoperative photograph demonstrating elevation of the cheek rotational fasciocutaneous flap (white arrow). The flap shows healthy tissue layers, buccal fat, fascia, and vascularized dermis, confirming flap viability.

**Figure 7:**
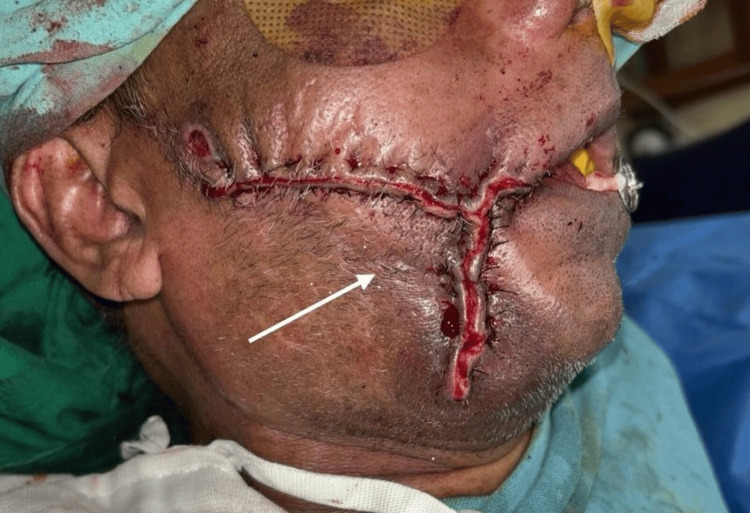
Immediate post-reconstruction intraoperative photograph demonstrating successful flap inset following right buccal mucosal advancement flap and cheek rotational fasciocutaneous flap. The white arrow indicates the inferolateral flap inset line. The characteristic rotation-advancement suture pattern (inverted-Y configuration) is visible across the right cheek with well-approximated margins, adequate tissue bulk, and no signs of vascular compromise at immediate post-operative assessment. Oral commissure architecture is preserved.

The immediate postoperative period was uneventful. Flap viability was confirmed intraoperatively by clinical assessment and handheld Doppler, monitored postoperatively at regular intervals with adequate color, turgor, and capillary refill at 24-hour and 48-hour reviews. The flap remained viable throughout the postoperative period with no evidence of vascular compromise. Nutritional support was continued post-operatively. No drain was placed intraoperatively. At the four-week follow-up, the patient demonstrated good wound healing, no dehiscence, no flap failure, improvement in mouth opening, and return to oral diet (Figure 8). Physiotherapy for progressive mouth-opening exercises was initiated to address residual trismus.

**Figure 8:**
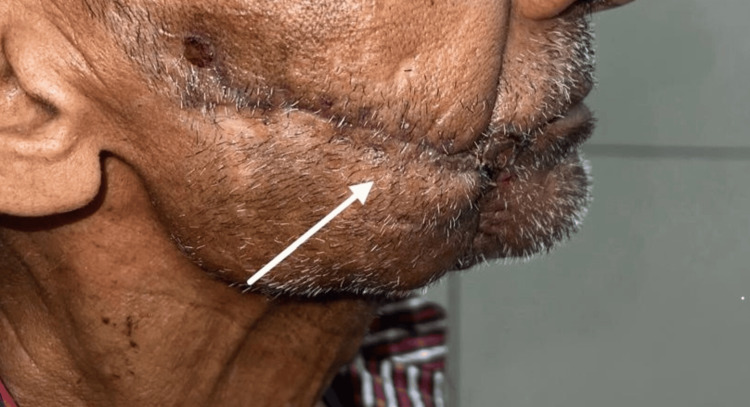
Postoperative follow-up photograph at four weeks demonstrating successful healing of the right buccal reconstruction. The white arrow indicates the well-matured flap scar along the cheek with complete epithelialization, preserved facial contour, and restoration of oral commissure position. The overall clinical appearance represents a satisfactory reconstructive outcome in an elderly immunocompromised host.

## Discussion

Cancrum oris (noma) is a rapidly progressive orofacial gangrene classically described in malnourished children aged between two and six years in sub-Saharan Africa, where untreated mortality approaches 90% [1, 5]. Our case departs from this classical profile in one critical respect-the patient was a 73-year-old adult. However, this departure is biologically coherent. Baratti-Mayer et al. established in a prospective case-control study that the true pathogenic determinants are immune dysfunction and protein-energy malnutrition, not age itself [11]. Both were unequivocally present in our patient: immune dysfunction evidenced by uncontrolled type 2 diabetes mellitus and malnutrition confirmed by serum albumin of 2.7 g/dl, confirming that elderly immunocompromised adults carry equivalent susceptibility to malnourished children and must be considered at risk. The diagnosis in this case is supported by all five WHO-defined criteria for noma: an orofacial gangrenous lesion, rapid tissue progression, fetid purulent discharge, a predisposing immunocompromised state, and the exclusion of alternative diagnoses [3]. Using the WHO noma staging system (2023), the patient presented with Stage 3 disease, active full-thickness gangrenous destruction of orofacial soft tissues with black eschar formation. Following staged debridement and wound stabilization, the case progressed to Stage 4/5, characterized by scarring, trismus, and functional sequelae necessitating reconstructive intervention [3]. The question of whether a preliminary diagnostic biopsy should precede surgical debridement in noma merits specific consideration. In the acute gangrenous phase, the biopsy of central necrotic tissue is non-diagnostic, yielding only necrotic debris without viable architecture; moreover, instrumentation of an actively spreading gangrenous field carries the theoretical risk of extending the infection plane.

Oral squamous cell carcinoma is the primary differential given the patient’s age and chronicity and the high baseline prevalence of tobacco-related oral malignancy in the Indian population. Features favoring noma over squamous cell carcinoma in this case included the acute onset, systemic sepsis, absence of mucosal induration, and lack of metastatic cervical lymphadenopathy. The polymicrobial wound flora; the characteristic transmural necrosis; acute necrotizing inflammation without epithelial atypia, invasive growth pattern, or perineural invasion; and the vascular thrombosis on histopathology are precisely those described by Enwonwu et al. as pathognomonic of Noma, collectively making the diagnosis unambiguous [6]. Rhinocerebral mucormycosis, a critical mimic producing an identical black orofacial eschar in immunocompromised hosts, was excluded by the absence of rhinosinusal opacification or bony erosion on CT and by negative Grocott-Gomori methenamine silver (GMS) and periodic acid-Schiff (PAS) staining for aseptate hyphae on histopathology. Cervicofacial actinomycosis was considered but excluded by the acute presentation, absence of woody induration and draining sinuses, and lack of sulfur granules or Gram-positive filaments on tissue staining. Necrotizing sialadenitis was unlikely given the absence of palatal involvement and parotid pathology and was excluded histopathologically by the absence of lobular salivary necrosis or reactive squamous metaplasia. Tuberculous ulceration was specifically considered in view of the patient's immunosuppressed status and Indian epidemiological context; however, the acute course, absence of constitutional symptoms, normal chest radiograph, lack of caseating granulomas on histopathology, and negative Ziehl-Neelsen staining collectively excluded mycobacterial aetiology.

Surgical management followed the two-phase protocol articulated by Adekeye and Ord et al. in their landmark series: staged debridement for source control, followed by deferred locoregional reconstruction [8]. Their observation that premature reconstruction in an infected wound uniformly results in flap failure directly informed our decision to perform three staged debridements prior to reconstruction, a decision vindicated by the satisfactory flap outcome in this case. The two-week debridement interval was not arbitrary but biologically determined: tissue demarcation between viable and non-viable tissue requires a minimum of five to seven days following each procedure, during which antibiotic therapy arrests spreading infection and early granulation signals the limit of necrosis. Premature re-debridement risks inadvertent excision of potentially salvageable tissue, while the interval between procedures permits systemic stabilization, nutritional repletion, and normalization of inflammatory markers, reducing cumulative anesthetic risk in this immunocompromised host. Each debridement was therefore performed not according to a fixed temporal protocol but in direct response to clinical wound assessment, with progression to the subsequent stage contingent upon confirmed infection arrest, advancing tissue demarcation, and systemic parameter improvement. The buccal mucosal advancement flap combined with a cheek rotational fasciocutaneous flap provided reliable two-layer coverage consistent with the reconstructive algorithm for moderate buccal defects, restoring oral mucosal lining and external cutaneous cover with tissue of comparable color and texture. 

Surgical intervention alone, however, was insufficient to ensure favorable outcomes; the success of staged reconstruction was contingent upon concurrent aggressive nutritional rehabilitation, given the profound protein-energy depletion that both predisposed to and was perpetuated by the gangrenous process. Nutritional rehabilitation was therefore central rather than adjunctive to our surgical plan [10, 11]. Baratti-Mayer et al. identified malnutrition as an independent risk factor for noma with an odds ratio of 8.3 [11]. Correia and Waitzberg independently demonstrated that hypoalbuminemia significantly increases surgical morbidity and impairs wound healing, findings directly applicable to our patient’s risk profile [12]. In our case, nutritional status was objectively monitored and guided by serial serum albumin measurements, which rose from 2.7 g/dL on admission to 3.8 g/dL preoperatively following targeted enteral nutritional rehabilitation, surpassing the minimum threshold of 3.0 g/dL considered necessary for acceptable flap healing outcomes in malnourished surgical patients. Reconstruction was deliberately deferred until this threshold was achieved, and the resultant improvement in wound healing capacity contributed directly to successful flap integration.

Underpinning both surgical and nutritional management was a targeted antibiotic strategy that evolved from empirical anaerobic cover to culture-directed therapy. This approach mirrored the WHO noma treatment framework [3] and the protocol described by Enwonwu et al. WHO emphasized that antibiotics function as a complementary rather than curative intervention, necrotic tissue creating an antibiotic-impenetrable anaerobic niche that only surgical debridement can eliminate [6]. Metronidazole-based anaerobic cover was initiated empirically at presentation, given its established efficacy against the obligate anaerobic polymicrobial consortium, specifically *Fusobacterium necrophorum* and *Prevotella *species, that drives the synergistic gangrenous destruction characteristic of noma, supplemented by broad-spectrum beta-lactam cover to address facultative aerobic organisms. Culture and sensitivity results corroborated the polymicrobial anaerobic profile; as isolated organisms demonstrated sensitivity to the empirical regimen, the antibiotic combination was subsequently rationalized to organism-directed therapy, with de-escalation from broad-spectrum cover reducing the risk of antimicrobial resistance selection and *Clostridioides difficile* superinfection, a particularly relevant consideration in this immunocompromised host on prolonged antibiotic therapy, ensuring microbiological control in parallel with wound bed preparation and nutritional optimization. Antibiotic therapy was continued for a total of 21 days, encompassing the perioperative debridement period and extending seven days beyond the final reconstructive procedure, in accordance with the principle of maintaining systemic anaerobic cover until wound bed sterilization was clinically confirmed.

As a single case report, findings cannot be generalized. Standardized wound scoring and prospective nutritional documentation were not performed. Long-term functional, cosmetic, and quality of life outcomes remain unassessed. Despite these limitations, this case report contributes to the sparse literature on noma in immunocompromised adults and illustrates a systematic clinicopathological and surgical approach that may inform future prospective series.

## Conclusions

Cancrum oris in the elderly, immunocompromised adult is a rare, diagnostically challenging, and potentially lethal condition with high mortality rates. This case demonstrates that a structured, multidisciplinary surgical approach, comprising prompt aggressive debridement, culture-directed antimicrobial therapy, rigorous perioperative nutritional support, and staged locoregional reconstruction, can achieve satisfactory functional and cosmetic outcomes even in this high-risk population.

Surgeons practicing in settings that manage immunocompromised or elderly patients must maintain noma in the differential diagnosis of progressive orofacial necrosis. Earlier recognition, timely surgical referral, and adherence to the principles outlined in this report are essential to reducing the burden of mortality and long-term disfigurement associated with this neglected but devastating disease.
